# Neonatal Thyroid-Stimulating Hormone Screening as a Monitoring Tool for Iodine Deficiency in Turkey

**DOI:** 10.4274/jcrpe.2526

**Published:** 2016-06-06

**Authors:** Nilgün Çaylan, Başak Tezel, Sema Özbaş, Nuran Şahin, Şirin Aydın, Deniz Acıcan, Bekir Keskinkılıç

**Affiliations:** 1 Public Health Institution of Turkey, Department of Child and Adolescent Health, Ankara, Turkey; 2 Public Health Institution of Turkey, Non-communicable Diseases and Cancer Vice Presidency, Ankara, Turkey

**Keywords:** Thyroid-stimulating hormone, screening program, Turkey, newborn

## Abstract

**Objective:**

Thyroid-stimulating hormone (TSH) level in neonates is recommended as an indicator for presence of iodine deficiency (ID) at a population level and as a monitoring tool in programs of iodine supplementation. The purpose of this study, based on data from the National Newborn Screening Program (NNSP) for congenital hypothyroidism (CH) in 2014, was to analyze neonatal TSH levels to predict the current status of iodine nutrition in Turkey.

**Methods:**

According to screening methodology, heel-prick blood samples of newborns were collected on filter paper cards usually on day 3-5 after birth (or shortly before discharge). Results of samples collected >48 h after birth were analyzed. The degree of severity of ID was assessed by using the epidemiologic criteria of the World Health Organization (WHO). Elevated TSH levels (>5 mIU/L) were processed and classified according to province, region, birth season, and sampling time.

**Results:**

A total of 1,298531 newborns were registered in the NNSP for the CH database. Of those, 1,270311 newborns had screening results collected >48 h after birth and were included in the statistical analyses. The national prevalence of elevated TSH was 7.2%. While the Gaziantep sub-region had the highest TSH elevation rate (15.9%), the Tekirdağ sub-region had the lowest rate (4.0%; p<0.001). Seasonal variations were also significant, and the elevated TSH prevalence rate was highest in winter (7.4%; p<0.001).

**Conclusion:**

National CH screening results suggest that Turkey may still be mildly iodine deficient. Nationwide studies should be performed for direct assessment and monitoring of iodine status in vulnerable populations to confirm accuracy of our results.

**WHAT IS ALREADY KNOWN ON THIS TOPIC?**

Iodine deficiency (ID) is one of the most prevalent deficiencies throughout the world and can cause brain damage in newborns. Thyroid-stimulating hormone level in neonates is recommended as an indicator of the degree of ID at a population level and as a monitoring tool in programs of iodine supplementation where a screening program is in force.

**WHAT THIS STUDY ADDS?**

According to the results of the newborn screening program for congenital hypothyroidism and using the World Health Organization guidelines, Turkey could be classified as mildly iodine deficient and iodine prophylaxis may be insufficient in vulnerable populations.

## INTRODUCTION

Iodine deficiency (ID) is one of the most prevalent deficiencies throughout the world and can cause brain damage in newborns; yet, it is easily preventable (1). Serious ID during pregnancy can result in a spectrum of morbidities referred to as ID disorders (IDD) including goiter, hypothyroidism, cretinism, mental retardation and delayed physical development, spontaneous abortion, stillbirth, congenital anomalies, and increased perinatal and infant mortality (1,2).

In order to prevent and treat IDD, universal salt iodization was adopted by the World Health Organization (WHO), the United Nations Children’s Fund (UNICEF), and the International Council for the Control of IDD (ICCIDD) in 1993 (3). Four major methods are recommended to assess and monitor the iodine nutritional status of a population: measurement of urinary iodine concentration (UIC), total goiter prevalence by palpation or ultrasonography, and measurement of serum thyroglobulin and thyroid-stimulating hormone (TSH) levels in neonates (4,5,6). TSH level in neonates is recommended as an indicator of the degree of ID at a population level and as a monitoring tool in programs of iodine supplementation where a screening program is in force (5,7,8).

In Turkey, the national IDD control program and mandatory salt iodization began in 1998. Before the initiation of the program, a survey was carried out between 1997 and 1999 and the median UIC in school age children (SAC) was found to be 25.5 μg/L, a finding which indicated presence of moderate ID (9). The main nationwide monitoring method of the program has been UIC in SAC; after implementation of the salt iodization program, the median UIC of SAC increased to 87 μg/L in 2002, to 117 μg/L in 2004, and to 130 μg/L in 2007 (10,11). Although these results suggest that iodine nutrition in the Turkish population has shown a gradual improvement, moderate to severe ID still exists in 27.8% of the Turkish population, mostly in rural areas (11).

The purpose of this study, based on the National Newborn Screening Program (NNSP) for congenital hypothyroidism (CH) 2014 database, was to analyze neonatal TSH levels to predict the current status of iodine nutrition in Turkey.

## METHODS

In Turkey, the nationwide screening program for CH was initiated in December 2006 by the Public Health Institute (PHI) of the Turkish Ministry of Health, in cooperation with a scientific committee consisting of members of universities and of governmental and non-governmental organizations. Since its initiation, both screening methodology and implementation of the program has gradually improved and the NSSP database has provided an opportunity to evaluate nationwide neonatal TSH levels (12).

Consistent with screening methodology, heel-prick blood samples from newborns were collected on filter paper cards (Whatman 903 filter paper) usually on day 3-5 after birth, or shortly before discharge if earlier discharge was planned. If the first sample was collected <48 h after birth, newborns were referred to a family medicine outpatient clinic for a second sample to be taken on day 3-5. For this manuscript, samples collected >48 h after birth were called timely samples and samples collected <48 h after birth were called early samples.

The filter paper cards were air-dried at room temperature and sent to one of the two laboratories of the PHI (in Ankara and İstanbul) for testing on either of two week days. These cards contained information on ID number, residence, birth province, contact address, date of birth, and date of sampling. The samples were tested within three working days after being received. TSH was detected with Trimaris neonatal TSH FEIA kits using the filter paper blood. Fluorescent enzyme immunoassay based on the TSH-specific two monoclonal antibody sandwich principle was used. The sensitivity of the TSH assay was 0.5-1.1 µIU/mL.

The degree of severity of ID was assessed by using epidemiologic criteria from the WHO (4). These criteria are based on the proportion of newborns with a TSH of >5 mIU/L whole blood: in iodine-sufficient areas <3%; mild 3-19.9%; moderate 20-39.9%, or severe >40% deficiency (4). Elevated TSH levels (>5 mIU/L) were processed and classified according to province, sub-region, birth season, and sampling time.

### Database

All personal information and screening data of newborns are registered in the NNSP database. Neonatal TSH screening data and other details were obtained from this system. All data were reviewed and statistical analyses were performed by the working group. Improbable records or those with missing descriptive information were excluded. Early screening results were analyzed separately. Results of samples collected >48 h after birth were used in the main statistical analyses.

### Statistical Analysis

Statistical Package for the Social Sciences (SPSS; Version 18.0) and Excel (Microsoft Office Excel 2007) software were used for data processing and statistics. The Kolmogorov-Smirnov test was used to determine normal distribution. Descriptive statistics were presented as mean ± standard deviation (SD) for normally distributed data, and as counts and percentages for categorical data. The relationship between the categorical variables was examined using the chi-square test. Student’s t-test was used for the comparison of two groups with normally distributed variables, and the Mann-Whitney U-test was used for abnormally distributed data. For the comparison of three or more groups, one-way analysis of variance (ANOVA) was used for normally distributed variables; otherwise, Kruskal-Wallis variance analysis was used. Results were evaluated with a confidence interval of 95%, and p<0.05 was considered statistically significant.

### Ethics

The parents of the babies tested were informed about the NNSP and heel-prick blood samples were only collected from live born babies after prior written consent from the parents.

## RESULTS

In 2014, 1,298531 newborns were registered with the NNSP for the CH database. Of those, 1,270311 newborns (97.8% of registered newborns; 51.3% boys, 48.7% girls) had timely screening results and were included in statistical analyses and 660946 newborns (50.1% of registered newborns; 51.0% boys, 49.0% girls) had early screening results. Mean sampling time was 7.3 days.

**Figure 1:**
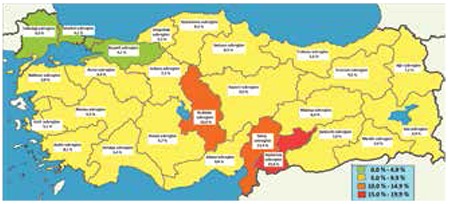
Elevated neonatal thyroid-stimulating hormone prevalence rates in 26 subregions of Turkey in 2014

**Figure 2:**
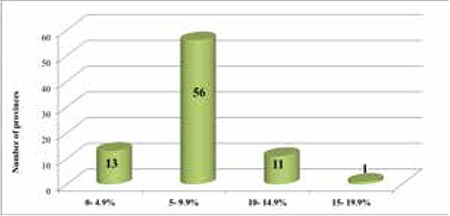
Distribution of the number of provinces according to elevated thyroid-stimulating hormone percentage figures

In 2014, the national prevalence of elevated TSH was 7.2%. Elevated TSH prevalence rates of all 26 sub-regions of Turkey were between >3% and 19.9% (in favor of mild ID) (Figure 1). While the Gaziantep sub-region, which is located in southeastern Turkey, had the highest elevated TSH rate (15.9%), the Tekirdağ sub-region, located in the north-western part of Turkey, had the lowest rate (4.0%; Figure 1). The difference between sub-regions was statistically significant (p<0.001). At the provincial level, Gaziantep had the highest (17.7%) and Tekirdağ had the lowest (3.2%) elevated TSH levels. The difference between provinces in terms of elevated TSH level was also significant (p<0.001). The distribution of the number of 81 provinces according to elevated TSH level is presented in Figure 2.

**Figure 3:**
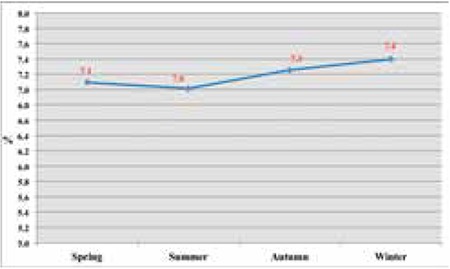
Seasonal variations of elevated neonatal thyroid-stimulating hormone levels

Samples drawn from the NNSP of Turkey were also assessed by birth season. Elevated TSH prevalence rate was highest in winter (7.4%). Seasonal variations were significantly different (p<0.001) and are presented in Figure 3.

**Figure 4:**
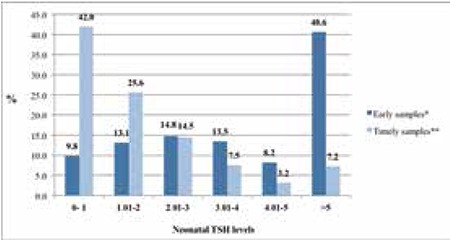
Cumulative frequency distribution of neonatal blood thyroid-stimulating hormone values according to sampling time. TSH: thyroid-stimulating hormone *Early samples: Samples collected <48 h after birth (n=660946) **Timely samples: Samples collected >48 h after birth (n=1,270311)

Elevated TSH prevalence rates of early samples were also analyzed and compared with the results of timely samples. While the elevated TSH prevalence rate of timely samples was 7.2%, the elevated TSH prevalence rate of early samples was 40.6%; this difference was significant (p<0.001). The cumulative frequency distribution of neonatal blood TSH values according to sampling time is presented in Figure 4.

## DISCUSSION

Historically, after the pioneering studies performed in Zaire and India where ID is endemic, neonatal TSH screening was recommended as a population monitoring tool for ID in addition to its role as a case-detection tool for diagnosing individual neonates with CH (7,8,13,14). This tool has been used to assess the severity of ID and also to monitor the outcome of iodine prophylaxis programs in countries or sub-national regions (7,15,16,17,18,19,20,21,22). Although some of them have provided conflicting results especially in cut off values (15,16), there are many successful country examples (17,18,19,20,21,22). In Thailand, with the application of a geographic information system to their neonatal TSH screening program, it has been possible to identify ID down to the sub-district level. Results of that study show that all provinces in Thailand suffer from ID at mild to moderate levels and the degree of severity increases year by year (17). In a study from southeast Poland, Tylek-Leman´ska et al (18) demonstrated that with the reintroduction of iodized salt in 1992, the prevalence of neonatal TSH results >5 mIU/L dropped from above 20% in 1991 to just over 5% between 1995 and 2000. The authors concluded that between 1985-2000, a drop in the incidence of IDD in newborns was clearly seen; furthermore, even low-grade iodine supplementation led to a significant decrease in TSH levels in newborns (18). In another study from Switzerland, Zimmermann et al (21) demonstrated that a 25% increase of iodine concentration in iodized salt resulted in a reduction of the neonatal frequency of TSH values >5 mIU/L from 2.9% to 1.7%, and iodine nutrition in children and pregnant women has improved from marginal to clearly sufficient.

Although the results of previous monitoring studies based on UIC in SAC have shown that Turkey is iodine replete, elevated TSH prevalence rates of the national CH screening results were in favor of mild ID (10,12). In Turkey, there are several previous regional studies based on CH screening and neonatal TSH levels (23,24,25,26,27). In a study on incidence of CH in the West Black Sea area (Bolu, Düzce, and Zonguldak provinces), elevated blood TSH (>5 mIU/L) concentrations were 26.7% and recall rate was 1.6% between 2000 and 2002 (24). In another study from Bursa province, between 1995 and 2004, Sağlam et al (25) reported a 5.8% recall rate and a 1/840 CH incidence, which could be explained by existence of ID. Another study on cord blood TSH of newborns showed a high frequency of elevated TSH concentrations (frequency of >10 mIU/L 28%) (26). In another recent study investigating the role of ID in the etiology of CH, ID frequency was 36% in CH patients and 88% in their mothers (27). Although these studies have differences in terms of methodology and study design, data based on regional CH screening also indicate that ID may still be a public health problem in Turkey.

In our study, according to the WHO guidelines, the lowest frequency for elevated TSH levels was detected in the northwest coastal areas of the country (Figure 1). This region is one of the most industrialized and urbanized areas of Turkey. Elevated TSH prevalence rate was highest in the inland areas and the south of the country (Figure 1). Furthermore, elevated TSH prevalence rate was high in winter compared to other seasons (Figure 3). The differences by region and season may be attributable to changes in regional and seasonal food preferences, differences in use of iodized salt, use of rock salt especially in local foods, and different agricultural practices.

The appropriate time of sampling for CH screening is between 48 h to 4 days; early sampling is not recommended due to the neonatal surge in the first 24 h after birth (28). Evidence shows that the mean TSH level in samples taken less than 24 h after birth was significantly higher than the mean TSH level of neonates after the first 24 h (29). In accordance with the recommendations, we found that elevated TSH prevalence rates of early samples were significantly higher than those of timely samples (p<0.001). Additionally, the cumulative frequency distribution of neonatal blood TSH values of early samples was considerably different from those of timely samples (Figure 4). In Turkey, both early heel-prick blood samples before discharge and day 3-5 heel blood samples were collected to achieve higher screening coverage by the end of 2014. Eventually, NNSP coverage reached 99% in 2014 and, due to the high costs and increased workload, early samples were stopped at the beginning of 2015.

Our study had some limitations. We had no data on maternal and newborn UIC and we could not correlate them with TSH results. Some factors other than ID (prematurity, birth weight, mode of delivery, maternal or newborn exposure to iodine-containing antiseptics, etc.) can affect newborn TSH levels (30). In this study, it was not possible to demonstrate the effect of these factors.

Finally, results of the national CH screening program suggested that iodine prophylaxis may be insufficient in vulnerable populations and we suggest that the following recommendations be taken into consideration in future work:

1- Nationwide studies should be performed for direct assessment and monitoring of iodine status in pregnant women, nursing mothers, and newborns in addition to systematic monitoring studies assessing the iodine status in SAC.

2- Maternal and newborn UIC and national neonatal TSH screening results should be correlated to determine whether neonatal TSH results can or cannot be used as a monitoring tool for the salt iodization program.

3- Regional and seasonal differences should be investigated.

4- New studies should be planned to determine factors affecting our national neonatal TSH levels other than ID.

## Ethics

Ethics Committee Approval: It was taken, Informed Consent: The parents of the babies tested were informed about the NNSP and heel-prick blood samples were only collected from live born babies after prior written consent from the parents.

Peer-review: External peer-reviewed.
